# NANDA nursing diagnoses associated with the occurrence of psychomotor agitation in patients with severe mental disorder: a cross-sectional study

**DOI:** 10.1186/s12912-023-01434-2

**Published:** 2023-08-28

**Authors:** María-Elena Garrote-Cámara, Raúl Juárez-Vela, Pedro-Manuel Rodríguez-Muñoz, Jesús Pérez, Juan-Luis Sánchez-González, Esther Rubinat-Arnaldo, Noelia Navas-Echazarreta, Teresa Sufrate-Sorzano, Iván Santolalla-Arnedo

**Affiliations:** 1 Department of Nursing, University of La Rioja, Logroño, La Rioja, Spain; 2 Faculty of Nursing and Physiotherapy, University of Salamanca, Salamanca, Spain; 3 Department of Psychiatry, University of Cambridge, Cambridge, UK; 4 Department of Nursing and Physiotherapy, University of Lleida, Lleida, Spain

**Keywords:** Nursing, Mental Health, Severe Mental disorder, Psychomotor agitation, Standardized nursing terminology

## Abstract

**Background:**

Psychomotor agitation is increased psychomotor activity, restlessness and irritability. People with psychomotor agitation respond by overreacting to intrinsic and extrinsic stimuli, experiencing stress and/or cognitive impairment. the aim was to analyse the association of nursing diagnoses with the disinhibition dimension, the aggressiveness dimension and the lability dimension of the Corrigan Agitated Behaviour Scale.

**Methods:**

This study was conducted in Spain using a multicentre cross-sectional convenience sample of 140 patients who had been admitted to psychiatric hospital units and had presented an episode of psychomotor agitation between 2018 and 2021.

**Results:**

The Corrigan Agitated Behaviour Scale was used to assess psychomotor agitation. Associated nursing diagnoses, violence directed at professionals and the environment are shown to be predictive values for the severity of the agitation episode. Moderate-severe psychomotor agitation episodes are shown as predictors of violence directed mainly at professionals and the environment.

**Conclusions:**

There is an urgent need for mental health nurses to have knowledge of the extended clinic in order to care for users and improve their health conditions in dealing with people, with their social, subjective and biological dimension.

## Background

Psychomotor agitation (PA) is a non-specific syndrome, with a multifactorial aetiology, in which there is an alteration of motor behaviour, with a disproportionate and disorganised increase in motor functions, which may present with vegetative activation (diaphoresis, tachycardia and mydriasis), severe anxiety, panic, lability, disinhibition, or other intense emotional states [1, 2].

The Diagnostic and Statistical Manual of Mental Disorders (DSM-5 Classification) of the American Psychiatric Association defines agitation as excessive motor activity associated with a sense of inner tension, the motor activity is usually non-productive, repetitive and consists of behaviours such as pacing, procrastination or fidgeting, hand wringing, tugging at clothing and inability to sit still [3]. At the first International Expert Meeting on Agitation, agitation was defined as “a state in which patients are unable to remain still or quiet, characterised by internal features such as motor hypersensitivity, racing thoughts and emotional tension; and external features, mainly motor and verbal hyperactivity, and impaired communication” [4], although not an exhaustive definition, it was developed for its usefulness for clinical practice. The patient will be in a state of mind sometimes associated with nervousness, anger, euphoria, unmotivated laughter, crying, and uncontrollable shouting, which may lead to verbal and/or physical aggression, a situation that poses a serious risk to the patient, family members, healthcare staff and/or environment. When the episode of PA is accompanied by aggression, it is usually reactive in nature, i.e. unplanned, occurring in response to a perceived threat and/or external provocation, usually accompanied by disinhibition, affective instability and/or high bodily arousal. The aetiology of PA can be classified into three groups; The first group includes pathologies of psychiatric origin (psychotic and non-psychotic disorders), a second group refers to disorders of organic origin (cranioencephalic trauma, epilepsy, intoxication, hypoxia, metabolic alterations, infectious processes of the central nervous system) and a third group of mixed processes (intoxication and/or abstinence due to substance use, such as alcohol, amphetamines, cannabis, opiates, anxiolytics, etc.) [3, 5, 6].

Psychomotor agitation is one of the most prevalent hospital emergencies in the care of patients with severe mental disorders, and its intervention must be carried out quickly and effectively, as patient safety may be compromised [5]. Various studies show that 2.6% of annual calls to emergency services in Spain correspond to psychiatric emergencies, of which 1.9% correspond to patients with psychomotor agitation [7]. Other studies report that 25% of patients with schizophrenia and 15% of patients with bipolar disorder suffer at least one episode of PA per year [8]. The multicentre cross-sectional STAGE study collected 7,295 psychiatric emergencies of which 4.6% were episodes of psychomotor agitation, 63% of patients were male and episodes occurred most frequently in patients with schizophrenia and bipolar disorder respectively; substance abuse was another common diagnosis, but rarely presented as a single disorder and was usually associated with a psychiatric diagnosis [9].

The management of psychomotor agitation, as well as its assessment, causes stressful situations for health professionals, posing a great challenge, so it is recommended to obtain information on various aspects, such as aetiology, and to establish a good differential diagnosis of the behaviour, before attributing it solely to a psychiatric origin [10]. One of the most widely used scales for the assessment of psychomotor agitation is the Corrigan Agitated Behaviour Scale [11], which provides both quantitative and qualitative data, determining the level of agitation and its characteristics in terms of the dimensions of lability, disinhibition, and aggressiveness associated with the agitation episode. This scale has been widely used in the assessment of psychomotor agitation in patients with severe mental disorders [12–14] and can be completed with the assessment of associated nursing diagnoses (ND), such as violence directed at professionals and the environment (00138) and violence to other patients and self-directed violence (00140), according to the North American Nursing Diagnosis Association - NANDA [15]. The ND is defined as a clinical judgement about the responses of the individual, family, or community to actual or potential health problems or life processes [16]. Determining the NDs associated with episodes of psychomotor agitation is essential in order to determine efficient nursing interventions to ensure quality care, offering clinical safety, in the environment and in the resolution of the case.

## Methods

### Aim

This study aims to analyse the association of nursing diagnoses with the disinhibition dimension, the aggressiveness dimension and the lability dimension of the Corrigan Agitated Behaviour Scale.

### Design

A multicentre, cross-sectional, convenience sampling was carried out.

### Scope of study

This study was conducted in the northern region of Spain (La Rioja), all data were collected by qualified nurses who had been specifically trained for this purpose, during the admission of patients to the psychiatric hospital units of the “BLINDED FOR REVIEW”.

### Study population

A sample of n = 140 participants was included.

### Inclusion criteria and exclusion criteria

The sample size was estimated according to the criteria for factor analysis with a minimum of 10 subjects for each item [17]. The Corrigan Agitated Behaviour Scale is an instrument of hetero-applied administration in which the interviewer must evaluate 14 items, which are quantified with a Likert-type scale according to the practitioner’s observation [12, 13]. The inclusion criteria were: patients diagnosed with a severe mental disorder, of both sexes, over 16 years of age, admitted to units of the mental health network of the “BLINDED FOR REVIEW”, and who developed an episode of psychomotor agitation between 2018 and 2021; exclusion criteria: not having any perceptual and/or cognitive impairment that prevents them from understanding the nature of their disorder, the clinical reasons for their admission or the objectives of this research and the data used in it.

### Variables and measuring instruments

Socio-demographic and clinical variables were analysed using descriptive statistics such as mean and standard deviation for quantitative variables and frequencies for categorical variables. In addition, other parametric techniques, such as median, standard deviation, skewness and kurtosis, were used to describe item responses and summarise the total scale score. The statistical association between qualitative variables is analysed using Pearson’s chi^2^ with a P < 0.05.

The Spanish version of this scale includes 14 items in three dimensions: disinhibition, items 1, 2, 6, 7, 8, 9 and 10; aggressiveness, items 3, 4, 5 and 14; lability, items 11, 12 and 13 [11, 14]. There are 14 items assessing the ability to sustain attention: impulsivity, pain tolerance and frustration management; cooperation and demands violence and threats; explosiveness, anger, and unpredictability; self-stimulatory behaviours; pulling the bed’s objects or restraints; wandering around treatment areas; restlessness and excessive movement; repetitive behaviours; excessive, rapid and loud language; mood swings; excessive crying and/or laughing; self-injurious behaviours. The nurse should score on a Likert-type scale of 4 degrees of intensity, from 1 (absence) to 4 (extreme degree). The sum of the scores of the 14 items determines the severity of agitation; the higher the score, the greater the severity. The score on each of the three dimensions determines whether the episode is characterised more by lability, disinhibition or aggressiveness [18, 19]. On the other hand, sociodemographic data of the patients, associated NANDA diagnoses and the main diagnosis leading to admission are also collected. In our study, we collected data on the following associated nursing diagnoses: risk of self-directed violence (00140) and risk of violence directed at others (00138), differentiating between violence directed at professionals, violence directed at other patients and violence directed at the environment.

### Statistical analysis

The statistical software used was SPSS [20].

### Ethical considerations

All methods were performed in accordance with the relevant guidelines and regulations. The information was treated confidentially and anonymously as they had dissociated data, following the Data Protection Regulation (EU) 2016/679 of the European Parliament and the Organic Law 3/2018. The researchers do not declare any ethical, moral or legal conflicts, nor do they claim to have received financial compensation of any other kind. Participants did not receive any type of compensation for answering the questionnaire, as it was voluntary and subject to informed consent. The study was approved by the “BLINDED FOR REVIEW” committee (reference “BLINDED FOR REVIEW”).

## Results

The main socio-demographic variables of the sample are presented below in Table 1. The gender distribution of the sample was evenly distributed, with 52.9% male vs. 47.1% female, with a mean age of 45.6 years. The majority of the sample (60.7%) had schizophrenia and other psychotic disorders as underlying pathology, with a slightly higher proportion of men (60%) than women (40%). In addition, 10.7% had a personality disorder, with a slightly higher proportion of women (60%) than men (40%). A small proportion of the sample had depression (9.3%), with a higher proportion of women (69.2%) than men (30.8%). Bipolar disorder was present in 8.6% of the sample, with a higher proportion of women (67.7%) than men (33.3%). Finally, mania was present in 2.1% of the sample, with 100% of women.

**Table 1:** Main socio-demographic characteristics of the sample (n = 140)

Variables			n	%
Age		< 18	1	0.7
		18–30	21	15
		31–50	69	49.3
		51–65	28	20
		66–79	17	12.1
		> 80	4	2.9
Sex		Women	66	47.1
		Men	74	52.9
Mental Illness				
	Schizophrenia and other psychotic disorders		85	60.7
	Depression		13	9.3
	Mania		3	2.1
	Bipolar		12	8.6
	Personality disorder		15	10.7
	Others		22	8.6

Table 2 shows the items of the Spanish version of the Corrigan Agitated Behaviour Scale (ABS), with values for mean, standard deviation, skewness and kurtosis. Most of the items are distributed normally, without excessive asymmetry and kurtosis. The highest scoring items are item 2 “Impulsive, impatient, poorly tolerates pain or frustration” and item 3 “Uncooperative, uncaring, demanding”. The lowest scores were recorded for item 7 “Pulls objects or restraints” and item 13 “Cries or laughs easily and excessively”.

**Table 2:** Items of the Spanish version of the ABS Corrigan Scale

Item	Mean	SD±	Asymmetry	Kurtosis
1. Poor attention span, easily distracted, inability to concentrate	2.8	0.8	-0.3	-0.3
2. Impulsivity, impatience, low tolerance to pain and frustration	3.2	0.8	-1	0.4
3. Uncooperative, does not allow care to be administered, demanding	3.1	0.8	-0.6	-0.2
4. Is violent, threatens people and property	2.9	1	-0.5	-0.8
5. Explosive with unpredictable fits of rage	2.9	0.9	-0.6	-0.6
6. They rock, groan, rub themselves, or show other self-stimulatory behaviours.	2.2	1.1	0.3	-1.2
7. Pulls the bed’s objects and restraints.	1.7	1	1	-0.4
8. Wanders through treatment areas	2.5	1.1	-0.1	-1.3
9. Restlessness that comes and goes, excessive movement	2.8	0.9	-0.5	-0.5
10. Repetitive motor or verbal behaviours	2.7	0.9	-0.3	-0.8
11. Speaks quickly, loudly or excessively	2.9	1	-0.5	-0.9
12. Sudden mood swings	2.8	1	-0.6	-0.7
13. Cries or laughs easily and excessively	2	1	0.6	-0.8
14. Is hurtful or insulting	2.7	1	-0.3	-1.1

The data for the standard deviation and median, as well as the minimum and maximum values of the total psychomotor agitation scale and its individual dimensions (disinhibition, aggressiveness and lability) are shown below (Table 3). The median (p50) of the sum total of the items of each of the dimensions of the scale was 37.4, indicating that on average psychomotor agitation was presented at moderate to high levels. With respect to the dimensions analysed, disinhibition had a medium prevalence and lability a medium to high prevalence. Aggressiveness was the most predominant characteristic during psychomotor agitation with a median score (p50) of 12.

**Table 3:** Standard deviation and median of the total level of psychomotor agitation and dimensions

Dimension	Median	SD	Min	Max
Disinhibition (1)	17.9	± 0.5	7	28
Aggressiveness (2)	11.6	± 0.3	4	16
Lability (3)	7.7	± 0.3	3	12
TOTAL	37.4	± 0.8	14	56

The total percentage of the intensity of psychomotor agitation (sum total of the items) and for each of the dimensions are described in Table 4. 90.71% of the patients with severe mental disorder reached moderate-severe intensity levels of psychomotor agitation (64.29% moderate and 26.42% severe), while 9.29% experienced lower levels. Aggressiveness was the dimension most associated with episodes of psychomotor agitation in patients with severe mental disorder; 40.71% presented severe levels of aggressiveness. The inhibition dimension and the lability dimension showed more moderate intensities of agitation.

**Table 4:** Intensity of psychomotor agitation

Dimension	Minor	Moderate	Severe
Disinhibition (1)	17.86%	60.71%	21.43%
Aggressiveness (2)	8.58%	50.71%	40.71%
Lability (3)	16.43%	61.43%	22.14%
TOTAL	9.29%	64.29%	26.42%

Table 5 shows the main NANDA nursing diagnoses listed in the Corrigan Agitated Behaviour Scale. In it, we find that both violence directed at other patients and self-directed violence (00140), with 27.1% and 17.1% respectively, are among the lowest percentages, while violence directed at professionals and the environment (00138) is among the highest percentages, with 36.4% and 35% respectively.

**Table 5:** Nursing Diagnoses (NANDA).

Nursing Diagnoses		n	%
**Violence directed at professionals (00138)**			
	YES	51	36.41
	NO	89	63.57
**Self-directed violence (00140)**			
	YES	24	17.1
	NO	116	82.8
**Violence directed at other patients (00140)**			
	YES	38	27.1
	NO	102	72.8
**Violence directed at the environment (00138)**	YES	49	35
	NO	91	65

The statistically significant association between the main nursing diagnoses and the different dimensions of the Corrigan Agitated Behaviour Scale is shown in Table 6. There is a statistically significant relationship between self-directed violence and the presence of the disinhibition dimension in episodes of psychomotor agitation (p = 0.016), mostly associated with severe levels. In relation to the aggressiveness dimension, there is a statistically significant relationship in each of the nursing diagnoses studied; the diagnosis of violence directed at professionals (p = 0.000), with predominantly moderate-severe levels of aggressiveness; violence directed at other patients (p = 0.041) shows predominantly moderate-severe levels of aggressiveness; and finally, violence directed at the environment (p = 0.014) shows predominantly moderate-severe levels of aggressiveness. In relation to the lability dimension, there is a statistically significant relationship in each of the diagnoses; the diagnosis of violence directed at professionals (p = 0.038) is associated with predominantly severe levels; self-directed violence (p = 0.012) is associated with moderate levels of the lability dimension; violence directed at other patients (p = 0.011) is mostly associated with moderate levels; the diagnosis violence directed at the environment (p = 0.012) is mostly present at moderate-severe levels.

**Table 6:** Nursing Diagnoses (NANDA) versus dimensions of the Corrigan Agitated Behaviour Scale

Disinhibition (dimension 1)	P	Minor	Moderate	Severe
• Violence directed at professionals	0,061	16%	40%	43.33%
• Self-directed violence	0,016	20%	10.59%	33.33%
• Violence directed at other patients	0,647	20%	29.41%	26.67%
• Violence directed at the environment	0,419	24%	36.47%	40%
**Aggressiveness (dimension 2)**	**P**	**Minor**	**Moderate**	**Severe**
• Violence directed at professionals	0,000	0%	28.17%	54.39%
• Self-directed violence	0,017	33.33%	8.45%	24.56%
• Violence directed at other patients	0,041	0%	25.35%	35.09%
• Violence directed at the environment	0,014	0%	33.80%	43.86%
**Lability (dimension 3)**	**P**	**Minor**	**Moderate**	**Severe**
• Violence directed at professionals	0,038	17.39%	44.19%	29.03%
• Self-directed violence	0,012	17.39%	12.79%	29.03%
• Violence directed at other patients	0,011	13.04%	36.05%	12.90%
• Violence directed at the environment	0,012	8.70%	38.37%	45.16%

## Discussion

Our study allows us to know the main nursing diagnoses associated with psychomotor agitation in patients with severe mental disorders. Severe levels of psychomotor agitation were recorded in more than 25% of the patients admitted and moderate intensity in more than 60%; these data are in line with the study carried out by Sacchetti et al., where the prevalence of severe psychomotor agitation was 23.7% and of moderate intensity 40.5% [21]. In the study by Hart et al., as in our study, higher levels of agitation are related to schizophrenia and psychotic disorders, in that study similarly, mainly young people and males [22]. Regarding the qualitative level, the disinhibition, aggression and lability dimensions assessed by the Corrigan Agitated Behaviour Scale show that the aggression dimension is the one most associated with psychomotor agitation in these patients; they experienced high levels of aggression during the agitation episode. Aggressive behaviour is defined as a natural adaptive behaviour that is intended to cause harm or pain, either physically or verbally, and is determined by cultural, environmental and social factors [23]. Through aggression, we are prepared for any kind of threat or stress that may pose a danger to our physical and/or psychological integrity, in order to preserve the survival of the individual [24].

The different situations throughout an individual’s life, understood as external factors that accumulate or are reinforced, will determine the individual’s actions, whether spontaneous or not, by means of neurological procedures that will determine them individually [25]. We can differentiate between two types of aggressiveness according to its origin; reactive aggressiveness is impulsive, affective, and hostile, produces an unpleasant emotional state, is unplanned, occurs when a threat and/or external provocation is perceived, and is normally accompanied by disinhibition, affective instability and high bodily arousal; and on the other hand, proactive, it is instrumental, motivated by the aggressor voluntarily, which is more related to violence and physical aggression, without causing remorse or repentance in the subject [26]. The study by Frauenfelder et al. shows that ND self-directed violence is more prevalent than violence directed at others, which differs from another study that assessed the prevalence of nursing diagnoses in inpatients in a psychiatric setting, which showed that ND violence directed at others was the third most prevalent ND [27]. Psychiatric environments are characterised by confinement from the outside world, isolation and containment, total observation and surveillance, as well as deprivation of autonomy, all of which can generate more exacerbated reactions leading to a risk of violence directed at others. Associated nursing diagnoses, violence directed at professionals and the environment are shown to be predictive of the severity of the agitation episode.

## Conclusions

There is an imperative need for mental health nurses to have knowledge of the extended clinic to attend to users and improve their health conditions in dealing with people, with their physical, psychological and social dimension. Nursing intervention in agitation is fundamental, this must be carried out quickly and efficiently, as patient safety is compromised, and must ensure quality care, clinical safety in the environment and the resolution of the situation. In the implication for clinical practice, having a high level of knowledge of the episodes of psychomotor agitation and their consequences makes it easier for the nursing professional to prevent the development of violent behaviour directed at professionals and the environment.

### Relevance for clinical practice

Psychomotor agitation is a state that presents in different psychiatric disorders. Identification and appropriate management are essential for the treatment of patients and in this regard, NANDA nursing diagnoses are a valuable tool for proactive care developed by nursing professionals. In addition, NANDA nursing diagnoses associated with psychomotor agitation can also be useful for the evaluation of the effectiveness of interventions, since, if specific care objectives are established for each nursing diagnosis, it is possible to evaluate whether the care interventions implemented are achieving the expected results.

With this research we hope to collaborate in the identification of patient health problems, specifically with severe mental disorder, in order to plan the care intervention determined for each case, thus contributing to improve the quality of care and the effectiveness of treatment.

### Limitations

Of the limitations present in the research, it is important to highlight that the sample used to obtain the results was obtained using a convenience method. The main reason behind this decision is related to the difficulty of gaining access to the target population, due to the complexity of working in a hard-to-reach environment and achieving voluntary participation of patients.

## Data Availability

The datasets used and/or analysed during the current study are available from the corresponding author on reasonable request.

## List of Abbreviations

PA: Psychomotor agitation
DSM: Diagnostic and Statistical Manual of Mental Disorders
ND: Nursing Diagnoses
NANDA: North American Nursing Diagnosis Association
ABS: Corrigan Agitated Behaviour Scale
